# *N*-Dihydrogalactochitosan Potentiates the Radiosensitivity of Liver Metastatic Tumor Cells Originated from Murine Breast Tumors

**DOI:** 10.3390/ijms20225581

**Published:** 2019-11-08

**Authors:** Chung-Yih Wang, Chun-Yuan Chang, Chun-Yu Wang, Kaili Liu, Chia-Yun Kang, Yi-Jang Lee, Wei R. Chen

**Affiliations:** 1 Radiotherapy, Department of Medical Imaging, Cheng Hsin General Hospital, Taipei 112, Taiwan; chungyihwang@yahoo.com.tw; 2 Department of Biomedical Imaging and Radiological Sciences, National Yang-Ming University, Taipei 112, Taiwan; cc1747@cinj.rutgers.edu (C.-Y.C.); arthur2600@hotmail.com (C.-Y.W.); kang31125@gmail.com (C.-Y.K.); 3 Biophotonics Research Laboratory, Center for Interdisciplinary Biomedical Education and Research, College of Mathematics and Science, University of Central Oklahoma, Edmond, OK 73034, USA; kliu9@uco.edu; 4 Cancer Progression Research Center, National Yang-Ming University, Taipei 112, Taiwan

**Keywords:** *N*-dihydrogalactochitosan (GC), metastatic tumors, radiosensitivity, triple-negative breast cancer, cancer stem cells

## Abstract

Radiation is a widely used therapeutic method for treating breast cancer. *N*-dihydrogalactochitosan (GC), a biocompatible immunostimulant, is known to enhance the effects of various treatment modalities in different tumor types. However, whether GC can enhance the radiosensitivity of cancer cells remains to be explored. In this study, triple-negative murine 4T1 breast cancer cells transduced with multi-reporter genes were implanted in immunocompetent Balb/C mice to track, dissect, and identify liver-metastatic 4T1 cells. These cells expressed cancer stem cell (CSC) -related characteristics, including the ability to form spheroids, the expression of the CD44 marker, and the increase of protein stability. We then ex vivo investigated the potential effect of GC on the radiosensitivity of the liver-metastatic 4T1 breast cancer cells and compared the results to those of parental 4T1 cells subjected to the same treatment. The cells were irradiated with increased doses of X-rays with or without GC treatment. Colony formation assays were then performed to determine the survival fractions and radiosensitivity of these cells. We found that GC preferably increased the radiosensitivity of liver-metastatic 4T1 breast cancer cells rather than that of the parental cells. Additionally, the single-cell DNA electrophoresis assay (SCDEA) and γ-H2AX foci assay were performed to assess the level of double-stranded DNA breaks (DSBs). Compared to the parental cells, DNA damage was significantly increased in liver-metastatic 4T1 cells after they were treated with GC plus radiation. Further studies on apoptosis showed that this combination treatment increased the sub-G1 population of cells, but not caspase-3 cleavage, in liver-metastatic breast cancer cells. Taken together, the current data suggest that the synergistic effects of GC and irradiation might be used to enhance the efficacy of radiotherapy in treating metastatic tumors.

## 1. Introduction

Cancer is a leading cause of mortality, decreased quality of life, and overwhelming stress for patients, their families, and our societies [1]. When cancer cells adapt to a tissue microenvironment distant form that in which the primary tumor originated, they lead to metastasis, a hallmark of malignant tumors. Cutaneous metastases are common in breast cancer. Metastatic breast cancer is relatively easy to diagnose, yet no effective method can totally cure it. Additionally, triple-negative breast cancer that does not express the genes for estrogen receptor (ER), progesterone receptor and human epidermal growth factor receptor 2 (HER2) is the most lethal and malignant breast cancer subtype, is notorious for its extremely high metastatic ability [2]. For instance, it has been reported that 50% of late-stage breast tumors will metastasize to liver [3]. Although a number of new therapeutic strategies have been developed in the past decades to combat metastatic cancers, including targeted therapy [4,5], hormonal therapy [6], and immunotherapy [7,8], the treatment of metastatic tumors remains the biggest challenge for researchers and clinicians.

Radiotherapy is a treatment approach using high-energy rays to kill cancer cells [9,10]. The intrinsic radiosensitivity of tumor cells is an important factor for determining the efficacy of radiotherapy. During tumor development, a decrease of radiosensitivity may allow tumor cells to escape from cell cycle arrest and apoptosis, even in the presence of DNA damage [11,12]. The development of radiosensitizers is an important pharmaceutical consideration for adjuvant radiotherapy. Radiosensitizers are designed to enhance the cellular responses to ionizing radiation, such as the reduction of the amount of intracellular thiol-related metabolites, the promotion of the formation of cytotoxic adducts, the suppression of DNA repair molecules, the interference of DNA replication, or the action of oxygen mimetics [13]. Unlike chemotherapy, ideal radiosensitizers should be biocompatible and have low cell toxicity [14]. This is important to obtain synergistic effects with radiotherapy.

*N*-dihydrogalactochitosan (GC) is a novel biocompatible immunoadjuvant for various clinical or preclinical cancer treatment modalities. For instance, laser immunotherapy (LIT) combined with GC administration was developed to induce a systemic antitumor immune response [15]. GC is synthesized by attaching galactose molecules to the free amino groups of chitosan, making GC water-soluble [16]. GC was originally developed for the stimulation of innate immunity, and it is not known if it possesses other biological properties, such as modulation of radiosensitivity in cancer cells.

In this work, we investigated the potential effect of GC on radiation-induced cell death in murine parental and sibling liver-metastatic 4T1 breast cancer cells. We studied the effect of GC on the levels of double-stranded DNA breaks, DNA damage marker γ-H2AX, and apoptosis—in terms of sub-G1 population and caspase-3 cleavage—upon radiotherapy. Our results showed the synergistic effects of GC and radiotherapy in the treatment of 4T1 tumors. The findings may lead to effective radiotherapy for the treatment of metastatic cancers, with enhanced effects resulting from the administration of GC.

## 2. Results

### 2.1. Isolation of Liver-Metastatic 4T1 Murine Breast Cancer Cells Based on Molecular Imaging

**Figure 1:**
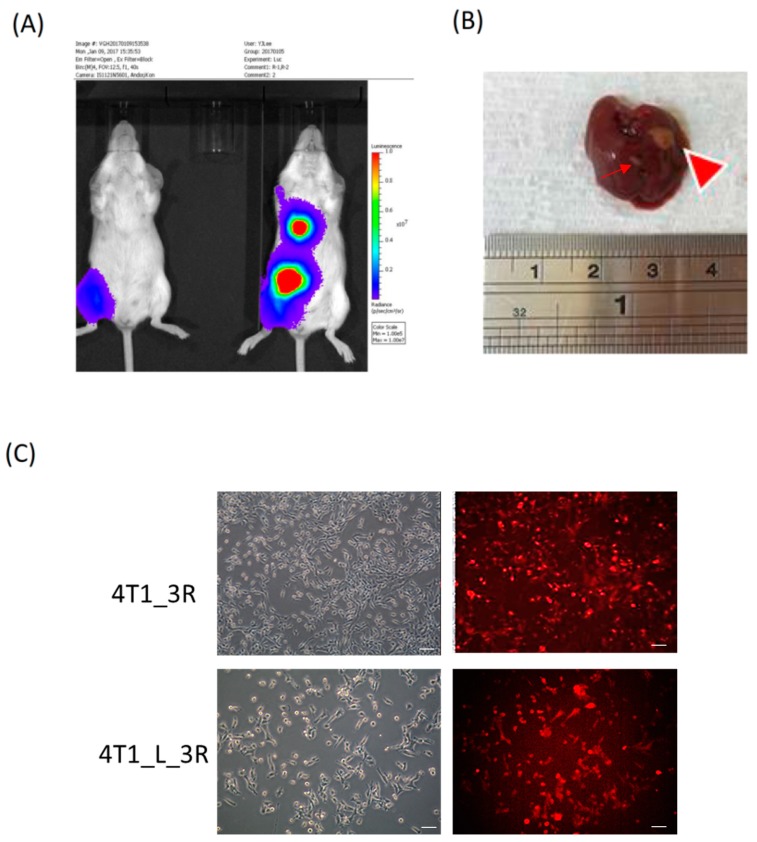
Tracking and isolation of liver-metastatic 4T1_3R breast cancer cells in Balb/C mice. (**A**) Coronal views of bioluminescent signals acquired at different time points after initial subcutaneous (s.c.) injection of 4T1_3R cells. (**B**) Resection of liver for the visualization of metastatic lesions. A major lesion (big arrowhead) and a minor lesion (thin arrow) were observed in the resected liver. (**C**) Fluorescence microscopic examination of red fluorescent protein (RFP) expression in isolated liver-metastatic 4T1_3R cells (4T1_L_3R) compared to original 4T1_3R cells. Scale bars = 100 μm.

Triple-negative 4T1 cells harboring the luciferase reporter gene (4T1_3R) were subcutaneously (s.c.) inoculated in the right thigh of Balb/C mice. After 3 weeks, bioluminescent imaging revealed robust signals around the abdomen and chest of the mice, while the signals were relatively lower in the first week (Figure 1A). We resected the liver from the tumor-bearing mice and observed the presence of metastatic lesion (Figure 1B). We subsequently isolated 4T1_3R cells from the resected liver and visualized the expression of the red fluorescent protein (RFP) reporter gene (Figure 1C). These cells were renamed 4T1_L_3R cells, corresponding to 4T1 triple-negative liver-metastatic tumor cells. The letter “L” was used to denote the tissue of origin, i.e., the liver.

### 2.2. Liver-Metastatic 4T1 Cancer Cells Exhibit Properties of Tumor-Initiating Cells

**Figure 2:**
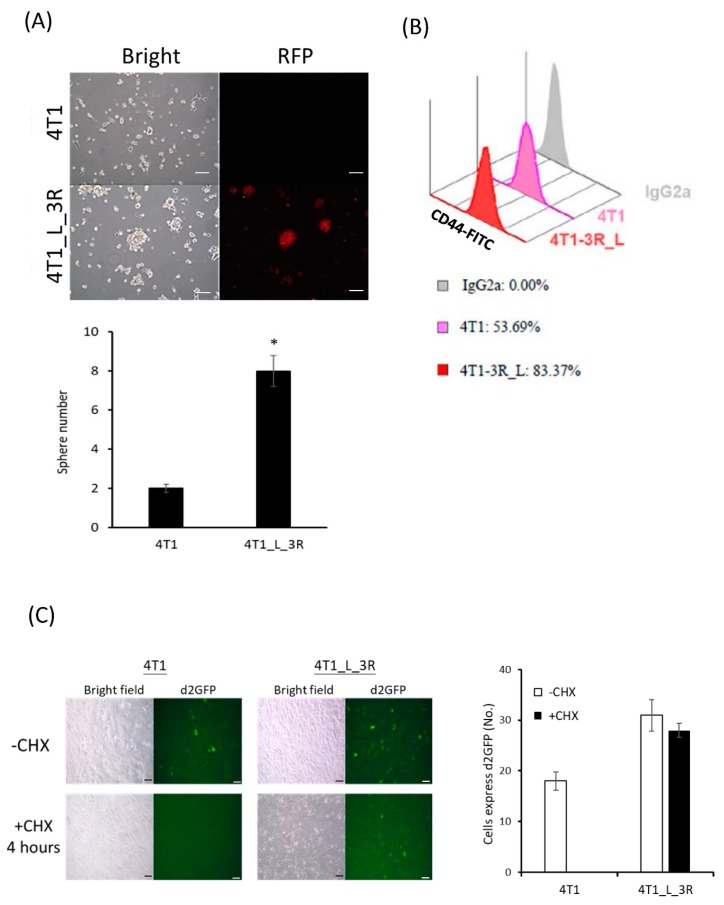
Characterization of liver-metastatic 4T1 cells for tumor-initiating properties. (**A**) Comparison of sphere-forming capacity between parental 4T1 cells and 4T1_L_3R cells. RFP expression remained detectable in the latter cells; * *p* < 0.05. (**B**) Flow cytometric analysis of CD44 marker expression in parental 4T1 cells and 4T1_L_3R cells. (**C**) Comparison of the degradation rate of d2GFP between parental 4T1 cells and 4T1_L_3R cells after treatment with cycloheximide (CHX) (50 μg/mL) for 4 h. Scale bar =100 μm.

We next examined if liver-metastatic breast cancer cells would exhibit tumor-initiating properties. The sphere formation assay showed that isolated 4T1_L_3R cells generated more sphere colonies than parental 4T1 cells (Figure 2A), indicating a higher number of tumor-initiating cells in liver-metastatic tumor cells than in parental 4T1-3R/non-metastatic cells. The expression level of CD44, a marker of breast cancer stem cells, was significantly increased in 4T1_L_3R cells compared to parental 4T1-3R cells (Figure 2B). As reduced 26S proteasome activity in cancer-initiating cells has been reported [17], we investigated protein abundance by using a short-half-life version of green fluorescent protein (d2-GFP) reporter gene to compare the accumulated protein expression levels in 4T1 cells and 4T1_L_3R cells (see Materials and Methods). The translation inhibitor cycloheximide (CHX) was added to the culture medium after the cells were transduced with the pd2-GFP plasmid. The results showed that: (1) d2-GFP was expressed in both 4T1-3R and 4T1_L_3R cells in the absence of CHX, ensuring that the introduced plasmid could function in this cell system and act as a positive control; (2) The expression of d2-GFP was diminished after CHX treatment in 4T1-3R cells, suggesting that degradation of expressed d2-GFP took place upon CHX treatment; (3) The fluorescent signal of d2-GFP could be detected in 4T1_L_3R cells, indicating that the protein degradation rate was lower than in 4T1-3R cells upon CHX treatment (Figure 2C). These data suggest that liver-metastatic 4T1 cells exhibited more tumor-initiating characteristics than parental 4T1 cells.

### 2.3. GC Enhances Radiosensitivity in Liver-Metastatic 4T1 Murine Breast Cancer Cells

**Figure 3:**
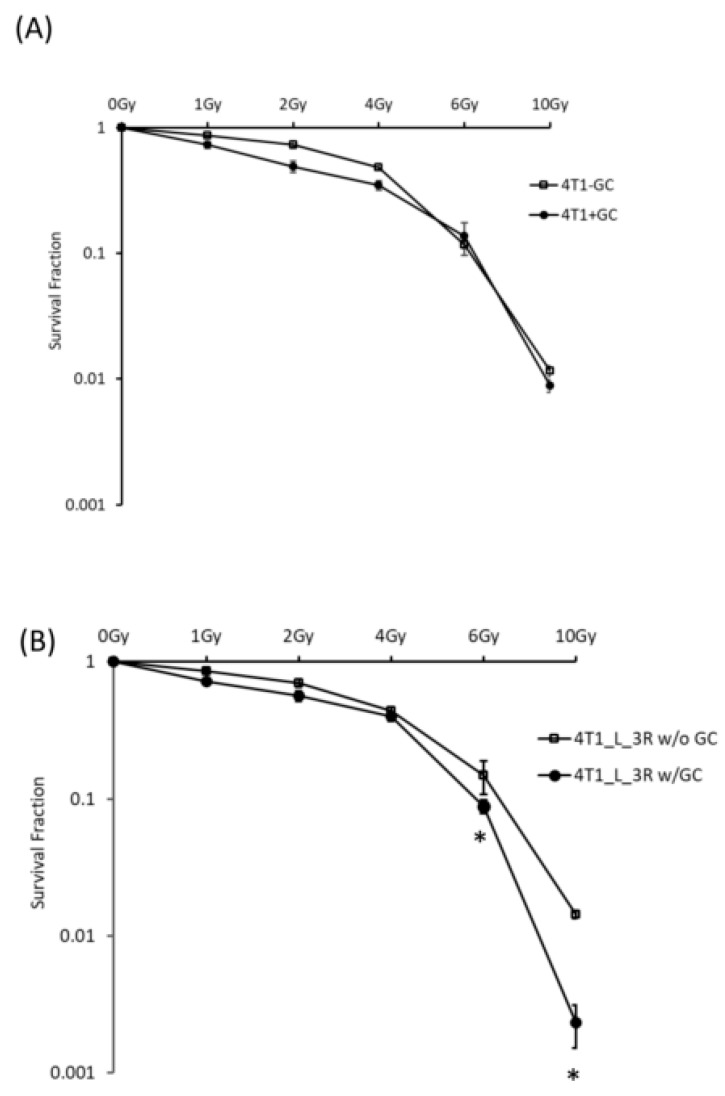
Analysis of survival fractions in cells exposed to X-rays with or without *N*-dihydrogalactochitosan (GC) pre-treatment. Cells were treated with 100 μg/mL of GC for 24 h before irradiation at different doses. The colony formation assay was used to compare the survival curves of (**A**) 4T1 cells and (**B**) 4T1_L_3R cell; * *p* < 0.05.

GC is a biocompatible immunostimulant that is known to boost the immune system against cancer growth [18]. GC has been used in combination with phototherapy, high-intensity focused ultrasound (HIFU), and radiofrequency ablation for the treatment of different types of tumors [19,20,21]. The effects of GC on the radiation responses of cancer cells has not been studied. Here, we compared the effects of GC–radiation combination on parental 4T1 cells and liver-metastatic 4T1 cells. The colony formation assay indicated that the survival rate of 4T1 cells was similar with or without GC treatment (Figure 3A), suggesting that GC does not significantly affect the viability of 4T1 cells without X-ray treatment. Interestingly, GC significantly enhanced the radiosensitivity of 4T1_L_3R cells after 6 Gy (Figure 3B), indicating that GC exerted a specific effect in the liver-metastatic cell type. These results suggest that liver-metastatic 4T1 cells are more sensitive to ionizing radiation in the presence of GC.

### 2.4. GC Enhances Ionizing Radiation-Induced DNA Damage in Liver-Metastatic 4T1 Cells

**Figure 4:**
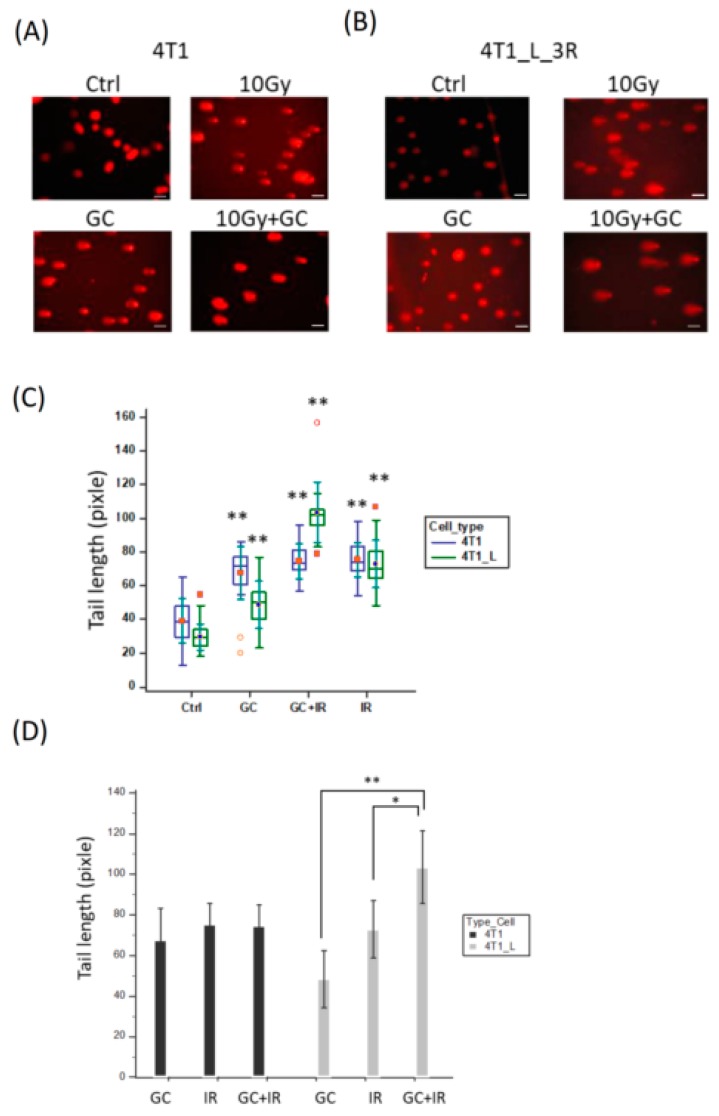
DNA damage analysis in cells exposed to X-rays with or without the GC pre-treatment. (**A**,**B**) Comet assay for parental 4T1 cells and 4T1_L_3R cells treated with GC, X-rays (10 Gy) or combined treatment, respectively. Scale bar = 50 μm. (**C**) Tail lengths determined by the comet assay were compared for untreated control, GC-treated, X-ray-treated, and combined treatment groups, and the results were analyzed using two-way ANOVA; ** *p* < 0.001. The data were presented as a box-and-whisker plot, where the central box represented the values from the lower to upper quartile (25 to 75 percentile). The middle line represented the median, and the dots in the middle position of the boxes represented central value markers. The far out values were displayed as open or solid circles. (**D**) Tail lengths were compared in cells subjected to combined treatment and individual GC or X-rays treatments. The results were analyzed using the *t*-test; * *p* < 0.05; ** *p* < 0.001.

Ionizing radiation is known to induce double-strand breaks (DSBs), but the DNA damage level may depend on the cell type, even when using the same radiation dosage [22]. Here, we used the single-cell DNA electrophoresis assay (SCDEA), also named comet assay, to analyze the levels of DNA damage in individual cells by visualizing the lengths of comet tails. Firstly, in parental 4T1 cells, the combination of GC and 10 Gy X-rays induced similar levels of comet tail lengths when it was compared with X-rays alone (Figure 4A). On the other hand, when we repeated the same experiment on 4T1_L_3R cells, we found that GC combined with X-rays resulted in longer comet tail lengths than X-rays alone (Figure 4B). The tail lengths of each experimental condition in these two cell types were analyzed by two-factor ANOVA, and the results demonstrated that GC-enhanced DNA damage upon radiation specifically occurred in 4T1_L_3R cells (Figure 4C). To further compare the combined effects of GC and X-rays with the effects of each individual treatment in 4T1 cells and 4T1-L-3R cells, we used independent-sample *t*-test for statistical analysis. The results showed that the combined treatment resulted in significantly elongated comet lengths than the individual GC or IR treatments (Figure 4D).

### 2.5. GC Combined to X-Rays Increases the Level of γ-H2AX in Liver-Metastatic 4T1 Cells More than in Parental 4T1 Cells

**Figure 5:**
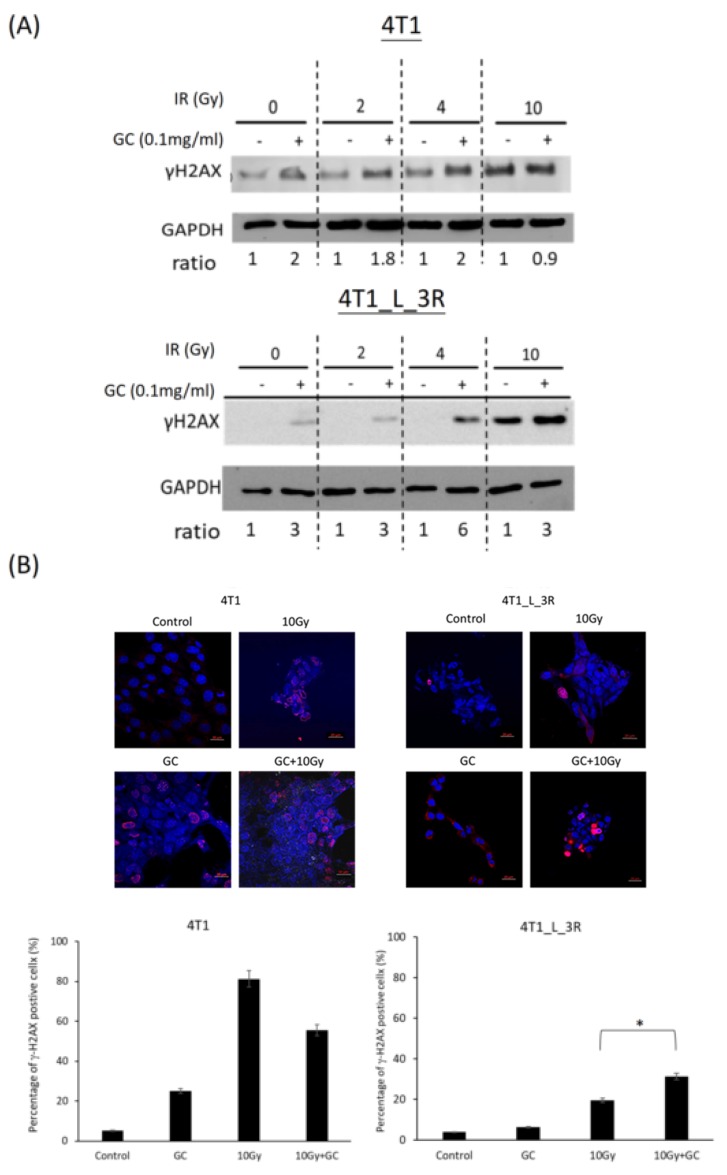
Effects of GC combined with different doses of X-rays on the expression of γ-H2AX. (**A**) Western blot analysis was used to detect the expression of γ-H2AX. The band intensity was quantified using densitometry, and the level of γ-H2AX was normalized to that of GAPDH. Effects of GC + X-rays on γ-H2AX were separately compared for different doses of X-rays. (**B**) γ-H2AX foci assay. The percentage of γ-H2AX-positive cells corresponds to the number of nuclei with γ-H2AX foci normalized to the total number of nuclei in each experimental group; * *p* < 0.05. Scale bar = 20 μm.

Since γ-H2AX is a biomarker of DSBs [23], we next compared the expression of γ-H2AX in parental 4T1 cells and liver-metastatic 4T1_L_3R cells after they were exposed to X-rays with or without GC treatment. The Western blot results showed that GC exhibited stronger effects on X-ray-induced γ-H2AX expression in 4T1_L_3R cells than in parental 4T1 cells for 2 and 10 Gy of irradiation (Figure 5A). Moreover, we performed a γ-H2AX foci assay for cells exposed to 10 Gy X-rays with or without GC treatment to validate the observations of the Western blot analysis (Figure 5B). The obtained results further suggested that GC combined with X-rays increased DNA damage.

### 2.6. Effects of GC Combined with X-Rays on Apoptosis of Liver-Metastatic 4T1 Cells

**Figure 6:**
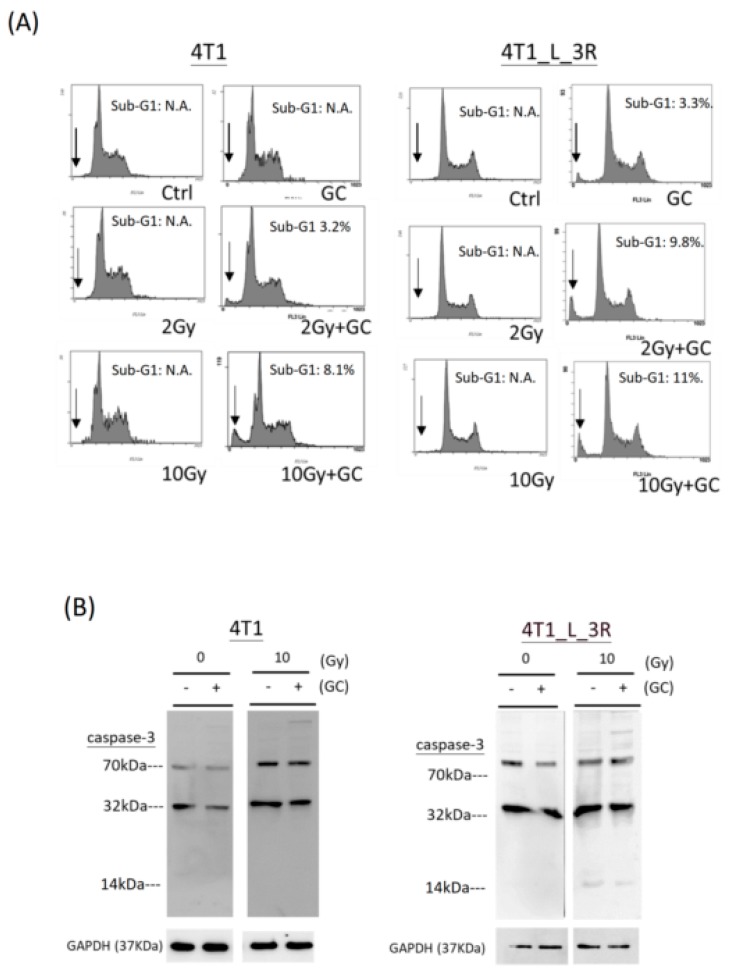
Effects of GC combined with different doses of X-rays on induction of apoptosis. (**A**) Analysis of sub-G1 population by flow cytometry. The arrows represent the peaks of sub-G1 population in the DNA histograms. The experiments were duplicated. N.A.: not available. (**B**) Western blot analysis of caspase-3 expression (~32 kDa) and cleavage (~14 kDa) in cells upon various treatments.

We next compared the level of apoptosis in parental 4T1 cells and liver-metastatic 4T1 cells after the GC + radiation treatment. The sub-G1 population and caspases-3 level were analyzed as the markers of apoptosis. It appeared that the combination of GC and 2 or 10 Gy X-rays induced a sub-G1 population (positions of peaks as indicated by the arrows) compared to control and radiation treatment in parental 4T1 cells and liver-metastatic 4T1 cells, and this effect was stronger in the latter cells (Figure 6A). As cells were collected 2 h after X-rays exposure (see Materials and Methods), we did not detect a significant change in cell cycle distribution in either cell type. On the other hand, we did not detect an increase of caspase-3 cleavage in both parental 4T1 cells and liver-metastatic 4T1 cells after they were exposed to combined GC and X-rays (Figure 6B).

## 3. Discussion

Metastasis is the most life-threatening condition in cancer patients because it is the main cause of cancer death. Metastatic malignancies re notorious for their extreme radioresistance and chemoresistance [24]. To date, there is no effective approach available to cure metastatic cancers. In our current work, we provide ex vivo evidence that GC can increase the radiosensitivity of metastatic breast cancer cells, therefore shedding light on its potential function as a radiosensitizer while serving also as an immunostimulant.

Accumulated literature suggests that metastasis is likely a property of cancer stem cells [25]. We have previously shown that remnant live 4T1 cells formed primary tumors with apparent necrosis and exhibited cancer stem cell-like characteristics [26]. Whole-body metastasis can also be detected at this stage [27]. Using in vivo bioluminescent imaging, we isolated metastatic 4T1 tumors in the liver and demonstrated that liver-metastatic 4T1 tumor cells displayed several characteristics of cancer stem cells compared to parental 4T1 cells (Figure 2B). We also previously found that 4T1 tumor cells could metastasize to a variety of organs, including brain, bone marrow, kidneys, lungs, and lymph nodes [19,26]. It is speculated that these organ-metastasizing 4T1 cells would exhibit cancer stem cell-related phenotypes as well. Therefore, the effects of different dosages of radiation on metastases in different organs are also worth being investigated in the future.

The survival fractions determined by clonogenic survival data were used to determine the radiosensitivity of the tested cells. The related survival curves can be compared using linear-quadratic (LQ) parameters (α and β). The α and β parameters represent irreparable and reparable damage, respectively [28]. An increase of the α parameter has been found for various radiosensitizing agents from the results of radiation dose–survival curves, and its contribution in clinical practice is promising, for example for fractionation radiotherapy [29]. In our experiments, the LQ analysis after plotting the fitted survival curves showed that the fitted α values of both parent 4T1 cells and liver-metastatic 4T1_L_3R cells were increased after GC treatment compared to the untreated controls. They were 0.076, 0.311, 0.109, and 0.252 for 4T1 without GC, 4T1 with GC, 4T1_L_3R without GC, and 4T1_L_3R with GC, respectively. Hence, GC was effective not only in 4T1_L_3R cells but also in 4T1 cells at low dose ranges, while it synergized with high doses of X-rays (6 to 10 Gy) in4T1_L_3R cells, increasing their radiosensitivity. As the LQ model is important for determining the fractions of radiotherapy, the current results suggest that the treatment of parental or metastatic murine 4T1 cells with combined GC and radiation should be clinically beneficial.

DSBs are mainly induced by ionizing radiation during cancer radiotherapy. In our study, the comet assay showed that GC combined with radiation raised the level of DNA damage in liver-metastatic 4T1_L_3R cells to a greater extent compared to ionizing radiation alone, and this effect was weaker in parental 4T1 cells. Technically, comet length was measured instead of tail moment because the shapes of the comets were too complex to be recognized by the quantitative analysis software (see Materials and Methods). Little is known about how GC influences the DNA properties to enhance radiation-induced DSBs. GC is derived from chitosan, and several lines of evidence have shown that chitosan can stimulate reactive oxygen species (ROS)-mediated cell death and interact with genomic DNA [30,31]. Similarly, GC has been reported to stimulate the formation of nitric oxide (NO), one of the reactive nitrogen species (RNS), in murine macrophage cells [32]. Whether GC will influence the ROS or RNS levels in cancer cells is unknown. It is of interest to further investigate if GC-enhanced radiosensitivity in metastatic cancer cells is due to changes in oxidative stress.

Radiation is known to induce the expression of p53, a genome guardian [33,34]. As 4T1 cells are p53-null [35], GC would enhance the radiation response through p53-independent pathways. Given that p53 is mutated in 50% of human cancers and is associated with radioresistance, the use of GC as an adjuvant in radiotherapy may be important for the treatment of p53-mutant cancers.

We observed that GC combined with ionizing radiation induced a sub-G1 population, used as an indicator of apoptosis, in liver-metastatic 4T1 cells but not in parental 4T1 cells. Radiation alone (10 Gy) did not influence the distribution in the cell cycle of liver-metastatic 4T1 cells and parental 4T1 cells. The samples were collected 2 h after X-rays exposure, which might be too short a time to observe cell cycle arrest. Nevertheless, caspase-3 cleavage was not increased accordingly. Although caspase-3 is a hallmark of apoptosis [36], caspase-independent apoptosis has also been reported [37]. This pathway is triggered and controlled by Bcl-2 family proteins, such as Bax and Bak proapoptotic factors, and mitochondria [38]. Therefore, whether the expression of Bcl-2 family proteins is affected by GC or GC in combination with irradiation should be investigated in the future.

Taken together, the current study provides evidence that GC enhanced the radiosensitivity of liver-metastatic murine breast cancer cells using an ex vivo model. It is of interest to further investigate whether GC would be beneficial as an adjuvant in radiotherapy to suppress the viability of aggressive metastatic tumor cells.

## 4. Materials and Methods

### 4.1. Cell Lines

The 4T1 triple-negative murine breast cancer cell line was purchased from ATCC (American Type Culture Collection, Manassas, VA, USA), and 4T1-3R cells harboring a PB-3R construct with multiple reporter genes (red fluorescence protein, firefly luciferase, HSV1-tk) was previously established as described [19]. 4T1-L cells represent the liver metastatic cells collected from BALB/c mice. Cell lines were maintained in RPMI (Life Technologies Inc., Carlsbad, CA, USA) medium with 10% fetal bovine serum, 50 μg/mL of penicillin/streptomycin, 2 mM of l-glutamine, were incubated at a 37 °C in a humidified incubator with 5% CO_2_, and passaged every two days. For ex vivo cell culture, liver metastasis tissues were cut into small pieces, seeded into 6-well plates, and maintained in RPMI medium with 10% of fetal bovine serum and 100 μg/mL of penicillin/streptomycin. The medium was changed every day to prevent contamination. Liver metastatic cells could be seen around one week and were further examined for the expression of red fluorescence using an inverted fluorescent microscope (Olympus CKX41, Olympus Co., Tokyo, Japan). The metastatic cells were collected and subjected to fluorescence-activated cell sorting (FACS, FACSAria, BD Biosciences, San Jose, CA, USA) for isolation of pure populations of metastatic cells from the liver.

### 4.2. GC and Ionizing Radiation Treatments

Cells were treated with 100 μg/mL of GC (Immunophotonics, St. Louis, MO, USA) for 24 h. For ionizing radiation exposure, cells were trypsinized and suspended into T-25 flask using an X-ray machine (RS 2000 Biological Research X-ray Irradiator; Rad Source Technologies, Inc., Suwanee, GA, USA) operating at 160 kVp and 25 mA. The dose rate from the source at a specific distance was 37.9 mGy/sec.

### 4.3. Establishment of a 4T1-3R Metastatic Animal Model

We used 4-week-old females BALB/c mice (*N* = 4) purchased from the National Laboratory Animal Center of Taiwan after 1 week of housing and quarantine for the establishment of a metastatic animal model. In total, 1 × 10^6^ 4T1-3R cells expressing a firefly luciferase gene were resuspended in 50 μL of OPTI-MEM and injected into the right leg of the mice subcutaneously, using a 27G needle. To monitor tumor growth and metastasis formation, fluorescent signals were detected by an In Vivo Imaging System (IVIS 50, Perkin Elmer Inc., Waltham, MA, USA) twice a week, and tumor sizes were measured by a caliper. The mice were sacrificed after metastatic lesions were confirmed by IVIS 50. Animal experiments were approved by the Institutional Animal Care and Utilization Committee (IACUC) of National Yang-Ming University, IACUC no. 1050422, 28 April 2016.

### 4.4. Colony Formation Assay

The cells were trypsinized and resuspended into T-25 flasks for radiation exposure at different dosages using an X-ray machine. The T-25 flasks were put on ice immediately after radiation exposure. Cells were then seeded into 6 cm dishes with technical triplicates and maintained in a humidified incubator without disturbance. After seven days of incubation, the 6 cm dishes were collected, washed with 1X phosphate-buffered saline (PBS) gently, and stained with 0.02% crystal violet for 10 min. The dishes were further rinsed and subjected to microscopic examination for quantification of colony number (each colony contained more than 50 cells). The plating efficiency was determined as the ratio of the number of colonies divided by the number of seeded cells. The surviving fraction was determined from the ratio of plating efficiency of the irradiated cells compared to that of the unirradiated controls.

### 4.5. Comet Assay

The evaluation of DNA damage was carried out by the comet assay, as previous reported [39]. In brief, approximately 10,000 cells were resuspended in PBS after radiation exposure and further mixed with 75 μL of low-melting-point agarose (LAMDA Biotech Inc., St. Louis, MO, USA). Multiple layers of gels were made on slides carefully, as described. Electrophoresis (25V, 40 min) was performed after incubation with lysis buffer, using an alkaline running buffer. To monitor DNA damage, the slides were incubated with 20 μg/mL of ethidium bromide and examined under a fluorescent microscope. A comet score software (CometScore 2.0. http://rexhoover.com/index.php?id=cometscore) was used to quantify the tail moment. At least 50 cells were counted in each group.

### 4.6. Protein Extraction and Western Blotting

The procedure of western blot analysis was performed as previous reported [40]. In brief, 30–50 μg of protein lysate was used to perform western blot analysis. The primary antibodies used in this study included anti-Histone H2A.XS139ph (GTX628789; GeneTex, Inc., Irvine, CA, USA), anti-caspase-3 (GTX110543; GeneTex, Inc.), and anti-GAPDH (MA5-15738; Thermo Fisher Scientific, Waltham, MA, USA). The enhanced chemiluminescent (ECL) kit (Bio-Rad Laboratories, Hercules, CA, USA) was used for the imaging together with ImageQuant LAS4000 (GE Healthcare, Buckinghamshire, UK). The quantification of optical density was achieved by ImageJ software (National Institutes of Health, Bethesda, MD, USA).

### 4.7. γ-H2AX Foci Assay

The γ-H2AX foci assay was performed as described with slightly modifications [41]. In brief, 3 × 10^4^ cells were seeded on coverslips placed in 35 mm dishes and incubated for 48 h. Cells were treated with 100 μg/mL of GC for 24 h, 10 Gy X-rays, or a combination of both, followed by fixation using freshly prepared 4% paraformaldehyde in PBS and permeabilization using 0.1% Triton X-100. The cells were then incubated with the anti-γ-H2AX antibody (1:500, same as above) for 1 h. The secondary antibody was Alexa Fluor™ 594-conjugated goat-anti-mouse antibody (1:500, Thermo Fisher Scientific). Cell nuclei were counterstained by 4’,6-diamidino-2-phenylindole (DAPI) for 10 min. The stained cells were mounted and examined using laser confocal microscopy (Zeiss LSM880, Carl Zeiss Microscopy GmbH, Jena, Germany).

### 4.8. Flow Cytometric Analysis of DNA Histograms

DNA content was determined using flow cytometry. The cells were treated as mentioned above before they were subjected to flow cytometric analysis. For sub-G1 analysis, the cells were collected 2 h after X-ray exposure. Cells were then fixed in ice-cold 75% ethanol (10^6^ cells/3 mL) at 4 °C overnight. The fixed cells were then spun and treated with RNase A for 30 min at room temperature. After centrifugation, the pellets were resuspended in 20 μg/mL of propidium iodide and dripped through 3a 7 m mesh. The samples were subjected to the BD FACS Caliber system (BD Bioscience) equipped with an air-cooled argon laser excited at 488 nm. DNA contents were determined with the EPICS Profile (Coulter Electronics, Hialeah, FL, USA) bundled with the machine.

### 4.9. Statistical Analysis

The means between two groups were analyzed using the *t*-test; when analyzing over three groups, the means were compared by two-way analysis of variance (ANOVA). Statistical analysis was performed using MedCalc^®^ software Ver. 18.2.1 (Ostend). A *p* value lower than 0.05 (*p* < 0.05) indicated significant differences.
